# Service use of older people who participate in primary care health promotion: a latent class analysis

**DOI:** 10.1186/s12913-017-2122-6

**Published:** 2017-03-06

**Authors:** John A. Ford, Kalpa Kharicha, Caroline S. Clarke, Allan Clark, Steve Iliffe, Claire Goodman, Jill Manthorpe, Nick Steel, Kate Walters

**Affiliations:** 1 Department of Public Health and Primary Care, Norwich Medical School, University of East Anglia, Norwich, UK; 2 Department of Primary Care and Population Health, University College London, Royal Free Campus Rowland Hill St, London, NW3 2PF UK; 3 Centre for Research in Primary and Community Care, University of Hertfordshire, Hatfield, UK; 4 Social Care Workforce Research Unit, King’s College London, London, UK

**Keywords:** Health promotion, Primary health care, Health services for the aged

## Abstract

**Background:**

Recruiting patients to health promotion programmes who will benefit is crucial to success. A key policy driver for health promotion in older people is to reduce health and social care use. Our aim was to describe service use among older people taking part in the Multi-dimensional Risk Appraisal for Older people primary care health promotion programme.

**Methods:**

A random sample of 1 in 3 older people (≥65 years old) was invited to participate in the Multi-dimensional Risk Appraisal for Older people project across five general practices in London and Hertfordshire. Data collected included socio-demographic characteristics, well-being and functional ability, lifestyle factors and service use.

Latent class analysis (LCA) was used to identify groups based on use of the following: secondary health care, primary health care, community health care, paid care, unpaid care, leisure and local authority resources. Differences in group characteristics were assessed using univariate logistic regression, weighted by probability of class assignation and clustered by GP practice.

**Results:**

Response rate was 34% (526/1550) with 447 participants presenting sufficient data for analysis. LCA using three groups gave the most meaningful interpretation and best model fit. About a third (active well) were fit and active with low service use. Just under a third (high NHS users) had high impairments with high primary, secondary and community health care contact, but low non-health services use. Just over a third (community service users) with high impairments used community health and other services without much hospital use.

**Conclusion:**

Older people taking part in the Multi-dimensional Risk Appraisal for Older people primary care health promotion can be described as three groups: active well, high NHS users, and community service users.

## Background

Fostering a culture which enables older people to age healthily is a key priority for most societies. The concept of healthy ageing, “the promotion of healthy living as the prevention and management of illness and disability associated with ageing”, [1] is not the same as active ageing where the focus is on creating opportunities to enable people to engage in health enhancing activities [2]. However both are underpinned by the principles of successful health promotion. Health promotion enables older people to maintain their independence; [3] arguably the greatest priority for people as they age [4]. From a policy perspective, a key driver of health promotion programmes is to reduce health and social care use [5].

Without effective recruitment of patients most likely to benefit from health promotion, even proven effective interventions may be unsuccessful. McInnes and colleagues undertook a systematic review of older people’s experiences of falls prevention programmes [6]. They concluded that programmes that are not properly targeted risk poor participation and undermine cost-effectiveness. Similarly Skelton and Beyer, in a review of exercise programmes, argued that participation of people who are “too fit” or “too frail” may mean the programmes do not deliver optimal outcomes [7].

General practitioners (GP) have frequent contact with older people, with 90% of NHS activity taking place in primary care, [8] and may be able to introduce health promotion initiatives into their practice [9]. This is supported by research that has found community-based multifactorial interventions help older people live safely and promoted independence [3]. An example of a primary care health promotion tool for older people is the Health Risk Appraisal for Older people (HRAO). This lifestyle risk assessment tool provides individualised written feedback to patients and their GPs and had the potential to reduce mortality when followed up by health advice in primary care [10, 11]. The HRAO has been expanded to include social, economic and environmental factors in the Multi-dimensional Risk Appraisal for Older people (MRAO) [12].

Avoiding unnecessary service use is a key focus for primary care health promotion programmes for older people, but no research has explored service usage among older people who take part in health promotion programmes. Our aim was to identify and describe groups of older people who took part in a primary care health promotion programme based on their service use, with a view to enabling better targeting of resources.

## Methods

We invited a random sample of 1 in 3 community-dwelling older people (≥65 years old) from five general practices in London and Hertfordshire to participate in the MRAO [12, 13] which was contextualised to each locality in 2012. The sampling strategy was based on a recruitment target of 150–200 per practice, equivalent to a 40% response rate. Care home residents, those with severe incapacitating/terminal illness and those unable to provide informed consent were excluded. Postal questionnaires were sent to all eligible participants, with one reminder sent to non-responders. Large-print questionnaires, translated versions and telephone assistance were available.

### Data collection

Baseline data collected included socio-demographic characteristics, well-being and functional ability, lifestyle factors, housing tenure, community engagement and service use. Service use was divided into seven categories and related to use within the past three months, as shown in Table 1.

**Table 1:** Categories of service use

Category	Service use included
Secondary care	Admittance to hospital, outpatients appointments or Accident and Emergency use
Primary care	GP or nurse consultations either at the practice, over the phone or at home
Community health care	Physiotherapist, osteopath, chiropractor, optician, dentist, podiatrist, audiologist, counsellor or smoking cessation ﻿service use
Paid care	Paid washing, cooking, meals or home help
Unpaid care	Unpaid washing, cooking or home help
Leisure activities	Library, sports activities, University of the Third Age, faith participation or education classes
General local authority services	Contacting the council about roads, refuse, housing, the environment, crime or carer services; using community or local authority transport, lunch clubs, day centres or community centre; contacting the Citizen’s Advice Bureau

Education attainment was measured as age at which a participant left school, dichotomised to up to 17 years or above. Alcohol use was measured using the AUDIT-C score [14]. Loneliness was measured using the de Jong Gierveld score [15] more than 1 on the 6-item scale or a self-reported feeling of loneliness much of the time. Health and functional status was measured using the mental and physical components of the SF12, [16] number of health conditions and basic activities of daily living. The extent of a participant’s social networks was assessed using the Lubben Social Network scale [17].

### Statistical analysis

Latent class analysis (LCA) was used to identify groups of primary care health promotion service users according to their common service use features [18]. Groups were generated based on use of the seven service use categories above, clustered at GP practice level. Within the LCA, binary variables were used for each category. The number of groups chosen for the LCA was based on model goodness of fit, assessed by the Bayesian Information Criterion (BIC) and Akaike Information Criterion (AIC), and discussion within the research team. Each participant was placed in a group based on the highest probability of membership. Service use and the socio-demographic characteristics of each group were described and statistically significant differences between groups assessed using univariate logistic regression, weighted for membership probability and clustered by GP practice. Descriptors for each group were agreed through discussion with the research team and patient and public representatives. The patient and public representatives were long-standing collaborators on the study although they were not familiar with the LCA technique. Prior to agreeing the descriptors within the research team, the lead researcher (JF) met with the patient and public representatives to explain the methods and results in lay terms to allow them to contribute more fully to the interpretation within the research team. The LCA was undertaken in Stata 13 [19] using a LCA plugin [20].

## Results

The response rate was 34% (526/1550) with 447 (85%) participants returning sufficient data to enable inclusion within the LCA. 127 participants were from the first GP practice, 154 from the second, 65 from the third, 46 from the fourth and 55 from the fifth. LCA found that BIC and AIC increased as the number of groups increased. After inspection of the groups we concluded that three groups (A, B, C) offer the most meaningful interpretation with a good model fit.

Table 2 shows the pattern of service use across the three groups. Statistically significant differences in service use between the groups are unsurprising because differences generated the groups in the LCA. Table 3 shows the characteristics of participants in each group.

**Table 2:** Patterns of service use across the three groups

Variable	Group A (*n* = 154) “active well”	Group B (*n* = 121) “high NHS users”	Group C (*n* = 172) “community service users”	*P* value		
				Group A vs B	Group A vs C	Group B vs C
Secondary care, % (*n*)	0.0 (0)	99.2 (120)	26.7 (46)	NA	NA	NA
Primary care use, % (*n*)	42.9 (66)	99.2 (120)	82.0 (141)	NA	**<0.001**	NA
Community health care use, % (*n*)	16.2 (25)	65.3 (79)	77.9 (134)	**0.031**	**<0.001**	**0.050**
Paid care, % (*n*)	2.6 (4)	0.0 (0)	28.5 (49)	NA	**<0.001**	NA
Unpaid care, % (*n*)	0.0 (0)	16.5 (20)	20.3 (35)	NA	NA	0.454
Leisure, % (*n*)	57.8 (89)	56.2 (68)	71.5 (123)	0.821	**0.024**	**0.006**
General local authority, % (*n*)	9.1 (14)	6.6 (8)	39.5 (68)	0.780	**<0.001**	**<0.001**

The figures in bold represent statistically significant results at *P*<0.05

*NA* not applicable, *NHS* National Health Service, *n* number of participants

**Table 3:** Characteristics of participants in each group

Variable	Group A (*n* = 154) “active well”	Group B (*n* = 121) “high NHS users”	Group C (*n* = 172) “community service users”	*P* value		
				Group A vs B	Group A vs C	Group B vs C
65–74, % (*n*)	70.1 (108)	56.2 (68)	54.7 (94)	**0.004**	**0.019**	0.078
75–84, % (*n*)	26.0 (40)	39.7 (48)	33.7 (58)			
85+, % (*n*)	3.9 (6)	4.1 (5)	11.6 (20)			
Female, % (*n*)	48.1 (74)	52.1 (63)	57.6 (99)	0.826	0.073	0.696
BMI (mean, SD)	26.6 (5.1)	26.8 (4.0)	27.2 (5.4)	0.333	0.415	0.261
White, % (*n*)	87.0 (134)	86.8 (105)	81.4 (140)	0.881	0.151	0.803
Education >17 yrs, % (*n*)	28.6 (44)	33.1 (40)	51.7 (89)	0.128	**<0.001**	**0.007**
AUDIT C score (mean, SD)	4.1 (2.8)	4.0 (2.9)	3.4 (2.8)	0.330	**0.041**	**0.007**
Never smoked, % (*n*)	56.5 (87)	55.4 (67)	57.6 (99)	0.148	0.934	0.203
Ex-smoker, % (*n*)	39.0 (60)	35.5 (43)	36.6 (63)			
Current smoker, % (*n*)	3.9 (6)	6.6 (8)	4.1 (7)			
State pension only, % (*n*)	27.9 (43)	27.3 (33)	23.3 (40)	0.380	0.124	0.793
Feeling lonely or de Jong score =/>2, % (*n*)	34.4 (53)	43.8 (53)	47.7 (82)	0.428	**0.029**	0.127
Carer, % (*n*)	18.2 (28)	13.2 (16)	16.3 (28)	0.138	0.576	0.902
Employment, % (*n*)	13.6 (21)	9.9 (12)	9.3 (16)	0.381	0.132	0.385
SF12 mental (mean, SD)	55.3 (6.5)	52.1 (8.1)	52.2 (10.2)	**0.008**	**0.008**	0.852
SF12 physical (mean, SD)	48.8 (9.1)	40.2 (12.2)	42.1 (13.9)	**0.028**	**<0.001**	0.917
No of conditions (mean, SD)	0.9 (1.0)	1.5 (1.2)	1.4 (1.1)	**0.006**	**<0.001**	0.497
Disability (BADL), % (*n*)	1.3 (2)	8.3 (10)	11.0 (19)	0.123	**0.002**	0.113
Low physical exercise, % (*n*)	24.7 (38)	45.5 (55)	44.8 (77)	**0.015**	**<0.001**	0.718
Mod physical exercise, % (*n*)	39.0 (60)	24.8 (30)	27.3 (47)			
High physical exercise, % (*n*)	35.7 (55)	29.8 (36)	27.9 (48)			
Lubben social network (mean, SD)	19.7 (5.2)	19.1 (5.2)	18.3 (5.7)	**0.024**	**0.035**	0.221
Volunteering, % (*n*)	22.7 (35)	21.5 (26)	27.3 (47)	0.496	0.181	0.066
Lives alone, % (*n*)	15.6 (24)	23.1 (28)	34.3 (59)	0.100	**<0.001**	**0.019**
Ground floor flat/ lift, % (*n*)	4.5 (7)	5.0 (6)	11.6 (20)	0.434	**0.021**	0.477
Flat other, % (*n*)	6.5 (10)	11.6 (14)	6.4 (11)			
House, % (*n*)	77.9 (120)	74.4 (90)	74.4 (128)			
Bungalow, % (*n*)	8.4 (13)	8.3 (10)	7.0 (12)			
Transport difficulties, % (*n*)	5.2 (8)	12.4 (15)	18.6 (32)	0.358	**0.001**	**0.006**
Internet use, % (*n*)	61.7 (95)	57.0 (69)	69.8 (120)	0.600	0.305	0.062

The figures in bold represent statistically significant results at *P*<0.05

*BMI* body mass index

*SD* standard deviation

*BADL* basic activities of daily living

Group A, “active well” (*n* = 154, 34.4%), did not use any secondary care and were lower users of primary and community health care compared to Groups B and C. They had low uptake of paid and unpaid care and lower use of leisure and general local authority services compared to Group C. Compared to groups B and C this group was younger, had better physical and mental health, fewer health problems , undertook more exercise and had wider social networks. Furthermore this group was less educated, consumed more alcohol, had lower levels of loneliness and disability, and was more likely to live in a house than a flat, compared to Group C.

Group B, “high NHS users” (*n* = 121, 27.1%), were characterised by almost all using secondary and primary health care services with a high proportion using community health care. None of this group used paid care, but a higher proportion used unpaid care compared to Group A. There was a lower use of leisure and general local authority services compared to Group C. This group was older, less healthy, undertook less exercise and had smaller social networks compared to Group A. They were less educated, consumed more alcohol and were less likely to live alone or have transport difficulties, compared to Group C.

Group C, “community service users” (*n* = 172, 38.5%), had low secondary care but high primary and community health care use. They had higher use of paid and unpaid care, and leisure and general local authority services compared to Groups A and B. This group was more educated, consumed less alcohol and were more likely to live alone and have transport difficulties compared to Groups A and B. Compared to Group A, this group was more lonely, had increased levels of disability and was more likely to live in a flat than a house.

## Discussion

About a third of older people agreeing to take part in this primary care health promotion study were fit and active with low service use (Group A, active well). Just under a third, Group B (high NHS users), had high impairments with high primary, secondary and community health care contact, but low use of non-health services. The remaining third, Group C (community service users), had high impairments and used community health and other services without much secondary care use.

### Strengths and limitations

The sample size was relatively small increasing the likelihood of a Type II error, especially in the univariate regression analysis, leading to true associations being missed as the evidence does not reach statistical significance. A low frequency of service use meant that categories were analysed as binary rather than discrete variables meaning that participants accessing a service more than once were not differentiated in the analysis. Whilst it was a strength being able to include health and non-health service use, it was not possible to further differentiate some service use data, such as acute vs. routine secondary care use or use of individual general local authority services. Data were self-reported, opening the possibility of reporting error, although the likelihood of this was reduced by using a relatively short recall period (3 months). The relatively short recall period may have missed some long term monitoring of chronic diseases undertaken on an annual basis. The availability and design of services may have varied across the five different GP practices. To mitigate for this we clustered by GP practice in the LCA. Participants were recruited as part of a research study and were required to give initial informed consent to participate. The results may therefore not necessarily reflect recruitment patterns or be generalizable to health promotion programmes used in practice.

The question of how to deal with membership probability in LCA has been debated at length [21–23]. The issue remains that assigning participants to a group based on the highest probability of assignment can lead to a participant with, for example, a probability of 0.33 of membership in Group A, 0.33 in Group B and 0.34 in Group C being allocated to Group C despite close similarity to other groups. To address this, we weighted the univariate regression on probability, meaning that participants with highest membership probability contributed more weight to the regression analysis.

### Comparison with existing literature

Whilst no previous research has used LCA to examine older people’s service use in health promotion programmes, common groups have been identified in specific services. Hastings and colleagues used it to explore older people’s emergency service use [24]. They identified five groups: “Primary Carederly” (39%, low hospital but high primary care use), “Wellderly” (34%, low use of any services), “Chronically Illderly” (14%, high primary care and hospital use), “Acute Carederly” (9.8% low primary care but high hospital use), “Sickest Elderly” (3.2%, highest emergency department visits). In our results Group A, active well, may be comparable to the “Wellderly” group, Group B, high NHS users, to “Chronically Illderly” and Group C “community service users” to “Primary Carederly”.

The well documented inverse care law [25] states that those who need health care most receive it the least. We found that two thirds of older people participating in this health promotion programme (the high NHS users and community service users) had low physical activity and high disease or disability, suggesting they are most in need of health promotion. Health promotion programmes not targeting those most in need have been criticised for increasing health inequalities because of participation by more affluent, healthier people [26]. Within the concept of proportional universalism health promotion should be offered across the whole population but proportionate to the level of need [27]. Our study did not target those most in need for the initial MRAO risk appraisal, and excluded those with terminal illness, unable to consent, or living in care homes. The results of a risk appraisal such as this can be used to identify those most in need for more intensive support to engage in health promotion. In this context, need could be conceptualised as ability to benefit from health promotion rather than level of disease or disability but more research is needed to identify this group.

### Implications for practice

Recruitment of the ‘right’ patients to health promotion programmes is essential for optimal benefits to be delivered. From a health and social care system perspective this would involve recruiting those with high current and future service use who might need fewer services with early preventative lifestyle changes. Group B, high NHS users, had high impairments and high use of primary, secondary and community health services and is potentially the group for whom interventions to reduce secondary care use might be most effective. This group is already engaged with primary and community health services and more effective use of these services may reduce their hospital use, though not necessarily. Future health promotion programmes may improve health system efficiency by targeting this group.

Group C, community service users, had high impairments and community health and non-health service use, but low secondary care use. They were more educated and more likely to live alone. One explanation is that they are maintained in the community, e.g. can access and receive sufficient proactive and long-term care services to prevent acute decline. Alternatively this may reflect a group with a pattern of long-term conditions which are stable and/or well-managed, does not necessitate hospital use but does result in impairment with high community health and social care needs. Thirdly, it may reflect a group who have a degree of poor health and risk of loneliness with high health literacy enabling them to navigate services resulting in high service use and participation in programmes, such as health promotion. Health literacy in this context would include the ability to obtain, read and understand information relating to health and non-health services to make appropriate decisions and follow instructions. For this group it is unclear if health promotion would sustain them in the community for longer or if programmes which facilitate more social interactions would be of greatest benefit.

### Implications for research

More research is needed to examine these groups in larger studies, current services, such as the NHS Health Checks, or different contexts. Furthermore research is needed to examine patient factors which lead to these groups through in depth qualitative studies or large cohort studies and to map future trajectories of service use, quality of life and mortality over time. This would help policy makers, public health and primary and social care commissioners to better target health promotion at those who would benefit most. More research is needed to develop health promotion programmes in older people which are targeted at those most in need and are acceptable to them. Ideally, these should be sufficiently generic to allow large scale implementation, but specific enough to accommodate personal and social factors. The on-going Home Health study is building on this work and looking to use personalised goal setting and behaviour change techniques in a community health and wellbeing promotion programme for older people with early frailty [28].

## Conclusions

We found that older people agreeing to take part in a health promotion programme in primary care could be described in three groups: active well, high NHS users and community service users. Effective recruitment of patients to primary care health promotion programmes is likely to be essential to increase efficiency and cost-effectiveness of service use.

## Abbreviations

AIC: Akaike information criterion
BADL: Basic activities of daily living
BIC: Bayesian information criterion
BMI: Body mass index
GP: General practitioner
HROA: Health risk appraisal for older people
LCA: Latent class analysis
MROA: Multi-dimensional appraisal for older people
n: Number of participants
NA: Not applicable
NHS: National health service
SD: Standard deviation
